# Supportive roles of brain macrophages in CNS metastases and assessment of new approaches targeting their functions

**DOI:** 10.7150/thno.40783

**Published:** 2020-02-10

**Authors:** Hua You, Szymon Baluszek, Bozena Kaminska

**Affiliations:** 1 Affiliated Cancer Hospital & Institute of Guangzhou Medical University, Guangzhou, China; 2 Laboratory of Molecular Neurobiology, Nencki Institute of Experimental Biology of the Polish Academy, Warsaw, Poland

**Keywords:** brain metastases, tumor microenvironment, cancer invasion, intracellular signaling, immune infiltrates, microglia, CNS border associated macrophages, perivascular macrophages, immunosuppression

## Abstract

Metastases to the central nervous system (CNS) occur frequently in adults and their frequency increases with the prolonged survival of cancer patients. Patients with CNS metastases have short survival, and modern therapeutics, while effective for extra-cranial cancers, do not reduce metastatic burden. Tumor cells attract and reprogram stromal cells, including tumor-associated macrophages that support cancer growth by promoting tissue remodeling, invasion, immunosuppression and metastasis. Specific roles of brain resident and infiltrating macrophages in creating a pre-metastatic niche for CNS invading cancer cells are less known. There are populations of CNS resident innate immune cells such as: parenchymal microglia and non-parenchymal, CNS border-associated macrophages that colonize CNS in early development and sustain its homeostasis. In this study we summarize available data on potential roles of different brain macrophages in most common brain metastases. We hypothesize that metastatic cancer cells exploit CNS macrophages and their cytoprotective mechanisms to create a pre-metastatic niche and facilitate metastatic growth. We assess current pharmacological strategies to manipulate functions of brain macrophages and hypothesize on their potential use in a therapy of CNS metastases. We conclude that the current data strongly support a notion that microglia, as well as non-parenchymal macrophages and peripheral infiltrating macrophages, are involved in multiple stages of CNS metastases. Understanding their contribution will lead to development of new therapeutic strategies.

## Introduction

Cancer develops in a heterogeneous tissue microenvironment, and surrounding or infiltrating non-malignant cells play important roles in creating a tumor niche and modulating anti-tumor responses 1. Tumor-associated macrophages (TAMs) accumulate in many cancers and activate multiple wound healing and tissue repair processes that are frequently associated with local and systemic immunosuppression 2-4. Several excellent reviews have presented many ways through which TAMs contribute to cancer progression 1-4. TAMs support tumor invasion by releasing an extracellular matrix (ECM) degrading proteases that contribute to reorganization of the surrounding tissues and facilitate cancer cell invasion. TAMs assist in cancer dissemination via the circulation or the lymphatic system. A majority of circulating cancer cells perish in the circulation, only a fraction of about 0.1% survives and less than 0.01% is capable of forming secondary lesions 5. Specific features of a small surviving subpopulation of metastasis-initiating cells are a subject of intensive studies. Perivascular macrophages support formation of intravasation sites where cancer cells spread into circulation and promote tumor angiogenesis. Some macrophages may co-migrate with cancer cells to a pre-metastatic niche and promote local remodeling of ECM. The presence of macrophages in the clusters of circulating cancer cells may protect them from the immune system 6.

**Figure 1:**
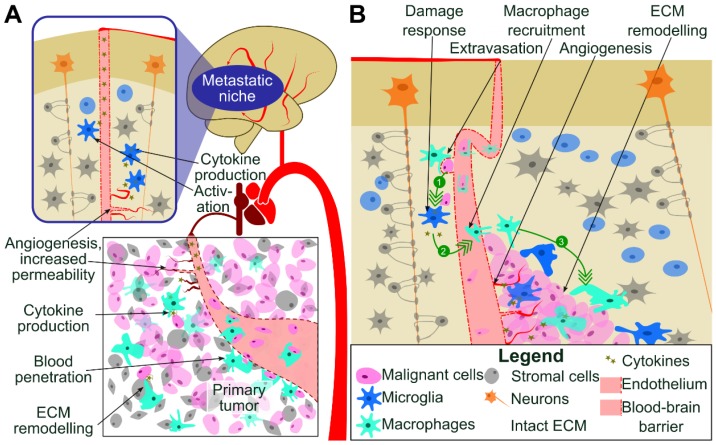
** Colonization of CNS by cancer cells from a periphery.** (A) Metastatic cancer cells from a periphery colonize CNS via penetration to the blood stream, hematogenous spread, extravasation from blood vessels, seeding a niche followed by tissue remodeling and growth of a secondary cancer. Those processes result in disturbance of CNS homeostasis. (B) Perivascular macrophages and circulating macrophages aid in extravasation of cancer cells (1). Subsequently, metastatic cancer cells secrete cytokines (2) activating CNS resident microglia and infiltrating peripheral macrophages to protect, repair, and instigate tissue repair. These events are associated with local immunosuppression, recruitment of microglia macrophages and tumor growth (3).

TAMs produce cytokines, chemokines and immunosuppressive molecules that participate in formation of the immunosuppressive microenvironment and contribute to the systemic immunosuppression 4,7. TAMs-secreted growth factors augment pro-survival pathways and may help tumor cells to resist cytotoxic chemotherapy 8. Colonization of CNS by cancer cells from a periphery is a complex process involving events such as extravasation from blood vessels, tissue remodeling and death of neurons. All those events result in disturbance of CNS homeostasis and elicit recuperating responses from microglia to protect, repair, and instigate the wound healing, associated with local immunosuppression (Figure 1). All these processes are actively assisted by microglia and infiltrating peripheral macrophages through mechanisms that are poorly characterized 9.

## 2. Brief characteristics of CNS macrophages

CNS is equipped with the resident, innate immune cells called microglia (located in the brain parenchyma) and non-parenchymal, CNS border-associated macrophages (BAMs) consisting of perivascular, meningeal and the choroid plexus macrophages, that are located at the brain-blood vessel interfaces, in the cerebrospinal fluid and in the choroid plexus, respectively. In the past those cells have been collectively called brain macrophages. The genetic lineage tracing approaches and single cell sequencing demonstrated that microglia and BAMs are transcriptionally distinct subpopulations and differ from the bone marrow (BM)-derived macrophages 10-12. Perivascular macrophages are located in the perivascular space surrounding arteries and veins penetrating deeply into the brain parenchyma, whereas meningeal macrophages are associated with the meninges. Perivascular macrophages express many surface proteins such as CD11b, CD45, CD68, CD115, CD206, CX3CR1, F4/80 and CD163, that are useful markers to identify these cells in flow cytometry and immunocytochemistry. CD163 is a membrane glycoprotein belonging to the scavenger receptor cystein-rich (SRCR) superfamily group B. Perivascular macrophages are positive for the phagocytic cell marker CD68 and the mannose receptor - CD206. In murine CNS, CD206 is strongly expressed on perivascular macrophages, and its expression is weak on infiltrating monocytes and microglia. CD163^+^ cells are positive for MHC class II antigens and the co-stimulatory molecules such as CD80, CD86, and CD40, suggesting their role in antigen recognition and presentation. CD163+ cells have higher expression of CD45 than microglia 12.

CNS macrophages behave as any other macrophages and function as phagocytic, antigen presenting and cytoprotective cells. Microglia, perivascular and meningeal macrophages originate from yolk sac myeloid progenitors, colonize CNS during early embryogenesis and persist throughout the entire life 13. Due to different ontogeny, location in CNS and highly specialized functions in a nervous tissue homeostasis and neuronal plasticity, microglia are distinct from peripheral macrophages 14. Microglia have numerous extensions and actively inspect the brain parenchyma and spinal cord. Microglia detect and remove damaged cells or apoptotic debris by phagocytosis, participate in adequate tuning of neural circuits and contribute to CNS homeostasis 15. Perivascular macrophages are important for preserving the integrity of the brain-blood barrier (BBB) 12.

Common immunohistochemical markers (Iba1, HLA-DR, Cd68, F4/80) detect all brain macrophages and have not been particularly effective in distinguishing resident microglia from BAMs and monocytes/macrophages invading human tumor tissues under pathological conditions. In flow cytometry, high CD45 expression can differentiate macrophages (Cd11b+CD45^high^) from microglia (Cd11b+CD45^low^). Recent studies using single cell sequencing and cell lineage tracing demonstrated that resident microglia are functionally distinct from BM-derived monocytes, which enter CNS under pathological conditions. Distinct transcriptomic profiles have been found in microglia and BM-derived macrophages infiltrating experimental gliomas and brain metastases in mice 16. The surface molecule CD49D, the α4 subunit of the integrin heterodimer α4β1 and Ly6C (lymphocyte antigen 6 complex, locus C1) have been proposed as good markers for flow cytometry to discriminate microglia and BM-derived macrophages in human brain tumors 16.

Our knowledge regarding heterogeneity and specific functions of brain macrophages within intracranial metastases is limited. Studying microglia-metastatic carcinoma interactions is hampered by: 1) a lack of convenient immunohistochemical markers distinguishing brain macrophages populations, 2) limited availability of patient samples, and 3) scarcity of appropriate animal models to study the microenvironment of CNS metastases. In this study, we describe the role of those cells in CNS invasion and metastatic spread based on available clinical and experimental observations. Several studies reported the accumulation of HLA-DR+ microglia/macrophages in the intracranial metastatic lesions in breast, melanoma, small cell lung, and non-small cell lung cancers 17, but understanding roles the specific subpopulation of brain macrophages play in metastatic seeding of CNS and cancer progression is incomplete. In the Table 1 we summarize conventional markers of main monocytic populations, their role in CNS biology under normal and pathological conditions 10-16, and potential roles in CNS metastases.

## 3. Characteristics of most common CNS metastases

CNS metastases, with incidence of 8.3 to 14.3 per 100,000 people 18, are recognized as the most common intracranial neoplasms and are found in autopsies of 20% cancer patients 19. The incidence varies between different tumor types; the most frequent are metastases of lung cancer (40-50%), breast cancer (20-30%), melanomas (20-25%), renal carcinomas (10-20%) and gastrointestinal tumors (4-6%). Predominant locations of CNS metastasis are the brain parenchyma and the leptomeninges. The incidence of CNS metastases has been increasing through recent decades due to multiple factors: increasing population age, prolonged survival of patients with primary and secondary advanced cancers, more effective detection of metastases with advancements in imaging techniques 20,21. New therapeutics targeting oncogenic kinases and immune checkpoint inhibitors increased overall and progression-free survival in patients, including those with brain metastases, but most of them did not effectively reduce metastatic burden and a majority of oncologic patients still die due to dissemination of the disease 22-24.

CNS metastases affect cognitive functions, speech, coordination, behavior, reduce the quality of life and ultimately lead to death. Standard of care for CNS metastases include local surgery, stereotactic radiosurgery or stereotactic fractionated radiotherapy combined with systemic chemotherapy. Despite the advancements in treating patients with metastatic cancer 22,23, a metastatic burden is cause of death in 90% of cancer patients 24. With modern treatments the median survival of patients with CNS metastases is 6 months 25, but the results vary depending on tumor histology, disease control, patient age and initial responses to a specific therapy 26. CNS metastases typically have a poor prognosis and patients survive 3-6 months, so consequently patients suffering from CNS metastases are sometimes excluded or are underrepresented in clinical trials of new drugs. Clinical trials in melanoma brain metastases with combined PD-1/CTLA-4 blockade showed ~50% intracranial response rate 27,28. Moreover, checkpoint inhibitors are considered effective as radiosensitizers in CNS metastases 29.

CNS metastases are relatively uncommon in children with the estimated frequency at 13% in autopsy studies and at 1.5% in clinical studies 30. CNS is frequently affected in pediatric leukemias and rare in solid tumors, where the occurrence is the highest in germ cell tumors, bone and soft tissue sarcomas 18,30. For leukemias and lymphomas, CNS invasion is carried by blood and spreads through the arterial and capillary system, or via direct expansion from the cranial bone marrow. The choroid plexus (a tissue with a dense network of capillaries) and connecting veins between the bone marrow and superficial arachnoid is suspected to be a site of cancer cell invasion into CNS 31.

Different cancers have varying propensity to form metastases in CNS. Non-small-cell lung cancer (NSCLC) is a most common in invading brain parenchyma (70% of metastases affect brain) and many patients with stage III or IV cancer (up to 55%) develop brain metastases in the course of the disease 32, with 3-5% patients having leptomeningeal metastases 33. A histological subgroup of small-cell lung cancer (SCLC) affects 20% of patients and is particularly aggressive. These metastases are treated with carboplatin, etoposide and preventive cranial irradiation. Despite recent advancement with anti-PD1 antibody atezolizumab, prognosis remains poor.

Breast cancer is molecularly classified by the expression of estrogen receptor (ER), progesterone receptor (PR) and human epidermal growth factor receptor-2 (HER-2). The triple-negative breast cancer is the most aggressive form. Its metastases to CNS are discovered in 46% of patients, but this reflects rather the overall metastasis burden than a specific property of this molecular subgroup. HER2+ breast cancers are thought to be particularly prone to cause delayed CNS metastases 34. In general, CNS metastases of breast cancer prognosticate better than to other sites (13.8 months) and respond to a systemic therapy in up to 80% of cases 35.

Metastases to CNS were found in 10-40% of melanoma patients, with the higher number of metastatic lesions (70- 90%) detected in brains during autopsy. Melanoma cells show some preferences as to location in CNS with the majority located within the frontal lobe (43.5%), less frequently in the cerebellum (8.6%) and rarely found in the hippocampus (<0.1%) 36. *BRAF* mutations occur in 40-50% of melanomas and treatments with specific inhibitors (e.g. vemurafenib, dabrafenib) were reported to be effective in a metastatic disease. The presence of *BRAF* mutation does not affect probability of CNS metastases, but a targeted treatment with vemurafenib decreases such probability 37. Melanomas are highly immunogenic tumors and checkpoint inhibitors have been very successful 38. Combining potent BRAF inhibitors with checkpoint inhibitors or stereotactic surgery have extended the therapeutic options for treating the brain metastases from melanoma 38.

Neurologic complications are common in leptomeningeal, epidural and brain parenchyma metastases of non-Hodgkin's lymphomas and are associated with a poor prognosis 39. Acute lymphoblastic leukemia (ALL) has a marked tendency to metastasize to CNS, it occurs in 5% of patients and ALL relapse in CNS predicts poor outcomes. CNS‐directed therapies such as: cranial irradiation, intrathecal chemotherapy and systemic administration of CNS‐penetrating chemotherapeutics, have reduced the frequency of disease recurrence 40. Spread of ALL rarely involves the parenchyma and is usually confined to the leptomeninges (lymphomatous meningitis).

Whole-exome sequencing of 86 matched brain metastases, primary tumors, and normal tissue examined if brain metastases harbor distinct genetic alterations from the ones observed in primary tumors. Most of the cases were derived from lung, breast and renal cell carcinomas. While all metastatic and primary sites shared mutational profiles suggesting a common ancestor, in 53% of cases, some alterations were found only in the brain metastases. Detected alterations were associated with the PI3K/AKT/mTOR, CDK, and HER2/EGFR signaling pathways and a sensitivity to pathway specific inhibitors in the brain metastases was proposed. Spatially and temporally separated brain metastasis sites were genetically homogenous, while distal extracranial and lymph node metastases were highly divergent from brain metastases 41. A recent TCGA (The Cancer Genome Atlas) study interrogating genomics of a tumor-of-origin and its metastasis among thousands samples of 33 tumor types revealed that metastases generally retained the mutational landscape of tumor of origin 42. It has become clear, however, that one of key features leading to metastasis formation is presence of a (pre)metastatic niche. Primary tumor secretome plays crucial role in this process. Exosomal micro-RNA alters microglia and BBB function which enables cancer invasion 43.

## 4. Supportive roles of brain macrophages in CNS metastases

### 4.1. Accumulation of microglia and macrophages in CNS metastases and impact on immune microenvironment

HLA-DR, Iba1 and CD68 are widely used as microglia and macrophage markers in a human tissue. HLA-DR is a heterodimeric cell surface glycoprotein comprised of a 36 kD α (heavy) chain and a 27 kD β (light) chain. It is expressed on microglia, monocytes/macrophages and can be weakly expressed on dendritic cells, B cells, and activated T cells. Iba1 is an ionized calcium binding adaptor molecule 1 and acts as a microglia/macrophage-specific calcium-binding protein with actin-bundling activity that participates in membrane ruffling and phagocytosis. CD68 is a member of the class D scavenger receptors and a glycosylated type I membrane protein that belongs to the lysosome-associated membrane proteins in macrophages. CD68 has been widely used as a pan-macrophage marker, although it can be weakly expressed on endothelial cells. In one of the first studies, the presence of brain macrophages in human CNS metastases was detected by immunohistochemistry (IHC) using an anti-CD68 antibody on paraffin-embedded tissue specimens of a small cohort consisting 17 metastatic tumors, including: lung, breast and clear cell kidney carcinomas. CD68+ macrophages were localized within the tumor tissue, at its periphery and its surroundings. In some cases, strongly stained CD68+ cells were visible in blood vessel walls. Those were likely perivascular macrophages. The study did not report any correlation between the type of tumor and extent of macrophage infiltration 44.

Further studies demonstrated Iba1+ cells with amoeboid, activated morphology found close to the cancer cells in lung cancer CNS metastatic lesions. Double labeling for the inflammatory markers revealed that a majority of those Iba1+ cells did not co-localize with either iNOS (inducible nitric oxide synthase) or TNF-α (tumor necrosis factor-α) staining, which can be interpreted as a lack of inflammatory, antitumor activation, and acquirement of the pro-tumorigenic phenotype 45. The number of CD68+ cells detected in metastases of lung adenocarcinoma was high and most positive cells displayed amoeboid morphology 46. Interestingly, the presence of activated brain macrophages correlated with the active immune microenvironment in SCLC brain metastases. Subsets of tumor infiltrating lymphocytes (TILs) such as CD3+, CD8+, CD45RO+, FOXP3+ and PD-1+ cells were analyzed in SCLC brain metastases and four matched primary tumor specimens. Patients with higher numbers of infiltrating CD45RO+ TILS survived longer. The expression of PD-L1 was detected on TILs and on the tumor infiltrating macrophages by immunohistochemistry 47. Similar proportions of TILs were detected in non-small cell lung carcinoma metastases 48. Detection of TIL infiltration was similar irrespectively of malignancy degree 48,49, which suggests an active immune microenvironment in those brain metastases.

Staining with the KiM1P antibody (recognizes CD68) showed positive cells in all samples of human breast cancer metastases to CNS and accumulation of amoeboid, positively stained cells in the boundary region between tumor and neighboring tissue. Their number varied from only few up to 50% of all cells 50. Immunohistochemical staining for a comprehensive panel of 21 inflammation-associated markers, including HLA-DR, in 17 human tissue specimens of brain metastases from breast cancer, NSCLC, SCLC and melanoma showed marked peritumoral accumulation and in some cases intratumoral infiltration of the HLA-DR+ microglia/macrophages. A high proportion of these cells showed a strong immunoreactivity for phagocytosis-associated markers, while a smaller subgroup of cells expressed molecules involved in production of free radicals. Only few B- and T-lymphocytes were observed in and around the brain metastases, and only a fraction of T-cells showed Granzyme B expression. Melanoma brain metastases had significantly less peritumoral brain macrophages than brain metastases of NSCLC did. The authors concluded that microglia/macrophages in brain metastases are activated and up-regulate proteins involved in phagocytosis, but activated cells do not activate the adaptive immunity 17. Altogether, the results clearly demonstrate accumulation of active (amoeboid) microglia/macrophages in CNS metastases of lung, breast cancers and melanomas. A few observations suggest that those cells did not show signs of the inflammatory, antitumor activation and rather adapt the pro-tumorigenic phenotype.

While most of the metastases were found in the brain parenchyma, metastases to the dura matter, one of the layers that cover the spinal cord and brain (meninges), were found during autopsy in 9-10% of all patients with different cancers 51. Neoplastic spread to leptomeninges is a result of cancer dissemination to the cerebrospinal fluid, which allows cancer cells to travel to multiple sites within the CNS, extravasate, and grow. Leptomeningeal metastases occur in 1-5% of patients with solid tumors and 5-15% of patients with leukemia 52. Melanoma, lung and breast cancers may form leptomeningeal metastases and a median survival in such cases is extremely short (2-3 months). Currently, there is no data on accumulation of microglia and macrophages in those tumors, but perivascular and meningeal macrophages due to their location may play a role in CNS metastases, as those cells are involved in immune-surveillance and participate in the recruitment of peripheral immune cells into the CNS in a response to pathological stimuli 53.

Primary central nervous system lymphoma (PCNSL) is a primary tumor but due to its location in CNS and interactions with local immune cells shares certain mechanisms with CNS metastases. The PCNSL is a rare form of lymphoma and accounts for 3%-4% of all primary brain tumors and 4%-6% of extra-nodal lymphomas 54. The majority of PCNSL is pathologically classified as diffuse large B-cell lymphoma (DLBCL) confined to CNS. While the genomic study of PCNSL samples using whole-exome sequencing and RNA-sequencing showed that PCNSL and DLBCL share some common gene expression and mutation profiles 55, DNA methylation profiles of the PCNSL are different from the systemic DLBCLs or normal lymph nodes, which suggests that PCNSL is a biologically distinct entity from the peripheral DLBCLs 56. Assessment of the phenotypes of myeloid cells was performed with a small PCNSL cohort (n=43), and numbers of CD68+, CD163+, and CD204+ TAMs were detected but without association with patient's prognosis 57. An independent study of a PCNSL cohort (n=47) showed the increased numbers of CD68+ cells to be significantly associated with progression-free survival, and a trend was observed for the increased abundance of CD163+ cells and a shorter survival. The IL-10 level in cerebrospinal fluids was correlated with infiltration of CD68 and CD163+ TAMs 58, and the diagnostic and prognostic value of IL10 in the cerebrospinal fluid in PCNSL was confirmed in other studies 59,60. The recent study, including the largest PCNSL cohort (n=114), showed that the increased number of CD68+ TAMs and indoleamine 2,3-dioxygenase (IDO) positive cells was associated with a favorable prognosis. The increased number of CD204+ cells and a high ratio of CD204+/CD68+ cells, indicative of the tumor-supporting polarization, were associated with a poor prognosis 61. *PD-L1* and *IDO1* were overexpressed by macrophage/microglia in PCNSL tissues, and gene expression profiling indicated that *IDO1* expression was positively correlated with the expression of macrophage and lymphocyte markers 61. PD-L1 expression on lymphoma cells correlated positively with the overall survival, whereas PD-L1 expression in the microenvironment showed a negative trend with the overall survival in a PCNSL cohort (n=64) 62. Osteopontin (encoded by the *SPP1* gene) was the most up-regulated gene (~10 fold) in PCNSL compared to non-CNS DLBCL 63. Osteopontin is a small phosphoprotein and an integrin ligand, which has been implicated in a variety of biological processes such as ECM adhesion and remodeling, cell migration, angiogenesis, proliferation, immunomodulation, chemotaxis of macrophages, dendritic cells, and T cells 64. Osteopontin may carry numerous interactions with stromal cells. The location of PCNSL cells in the perivascular spaces may indicate interactions of the malignant B cells with components of the blood-brain barrier: endothelial cells with up-regulated MHC class I and II antigens, ICAM-1, and vCAM-1, and perivascular macrophages.

### 4.2. Roles of microglia and brain macrophages in CNS metastases- lessons from the animal models

Cancer cell metastasis to CNS is modeled in animals by injection of rodent cancer cells (derived from spontaneous, chemically induced and genetically engineered tumors) and human xenografts via a carotid artery or directly to the brain of immunodeficient or immunocompetent rodents 65. While none of animal models fully reflects tumor progression observed in patients with a metastatic disease, various models provide important mechanistic insights into the metastatic process and allow testing potential therapeutics. Human breast cancer cells of different lines: MDA-MB-435, MDA-MB-231/brain, injected intracardially to immunosuppressed SCID mice and murine 4T1 cells injected to BALB/c mice formed tumors, although cancer cells needed more time to extravasate into the brain parenchyma than into other organs. The cancer cells were cleared from the brain microvessels and extravasated from day 3 to day 7 after injection, with exception of MDA-MB-231/brain cells, which were slower 66. Cells invading the brain parenchyma induced locally activation of astrocytes and microglia. Astrocytes up-regulated expression of GFAP (glial fibrillary acidic protein), Nestin or both. Microglia (detected with F4/80 staining) infiltrated into the breast cancer mass, accumulated in the surrounding gliosis zone, and formed contacts with cancer cells directly after successful extravasation 66,67. Microglia associated with cancer cells were heterogeneous and consisted of activated, hypertrophic microglia and reactive microglia with amoeboid cell morphology 66. When 99LN-BrM cells derived from MMTV:PyMT breast cancer cells were injected to syngeneic, immunocompetent mice with Cx3cr1-based myeloid cells (a lineage tracing model), both infiltrating microglia and BM-derived macrophages were detected in brain metastases 16.

Not only do microglia react to metastasis, it is also crucial for metastatic niche formation. Recently, role of CEMIP (cell migration-inducing and hyaluronan-binding protein) was shown to predict brain metastasis formation. It was selectively produced by cells metastasing to the brain but did not support metastasis growth. It rather upregulated inflammatory response in microglia 68. Microglia co-cultured with MCF-7 and 410.4 breast cancer cells *in vitro* and in living brain slice cultures (in which BM‐derived macrophages were absent) promoted cancer cell invasion and colonization of the brain tissue. Blocking microglia function with the bisphosphonate clodronate (an inhibitor of cells of a monocytic lineage) reduced cancer cell invasion. Stimulation of the TLR4 pathway shifted microglia to a pro-inflammatory and anti‐invasive phenotype. In organotypic brain slice cultures, microglia facilitated transport a single invading cell as well as cancer cell cohorts. Gene expression studies of microglia co-cultured with carcinoma cells did not show up-regulation of whole gene-set of the pro-tumorigenic (M2) phenotype but identified TLR and WNT signaling as the most affected pathways in those microglia. Both pathways participate in tissue regeneration and repair 50.

In brain metastases developing after intracarotid injection of different breast cancer cells (4T1, PyMT, or MDA-MB-231), CD11b+F4/80+ cells were detected by flow cytometry as the most abundant infiltrating immune cell population. The infiltration of myeloid-derived suppressor cells (MDSCs; CD11b+Gr1+), granulocytes (CD11b+Ly6G+) and monocytes (CD11b+Ly6C+) into dural metastases was greater than in parenchymal lesions 69. The T-cells (CD3e+) were rarely detected in either location. Of note, gene expression profiling revealed significant differences in gene expression of cancer cells that have metastasized to the brain parenchyma or the dura, with the high level of mRNA for Lymphotoxin β (LTβ) in parenchymal compared to dural metastatic lesions. The lower levels of *inos, MHCII, CD11c, arg1, ifnγ and tnfα* in CD11b+ cells from parenchymal versus dural metastasis were detected. The expression of *cd206* (a pro-tumorigenic phenotype marker) was significantly increased in parenchymal CD11b+ cells. This pattern of marker gene expression suggests that the parenchymal microglia/macrophages are more twisted towards the pro-tumorigenic phenotype compared to dural cells. It also confirms that a location of a metastatic site matters 69.

Signaling pathways underlying communication of cancer metastatic cells with microglia are similar to the ones operating in peripheral metastases and include Wnt/β‐catenin signaling, CXCR4 and its ligand SDF1 and PI3K pathway. Microglia‐dependent invasion of breast cancer cell co-cultures and in living brain slices was abolished by DKK‐2 (a secreted Wnt antagonist which antagonizes predominantly Wnt/β‐catenin signaling) 50. The *cxcr4* gene coding for C-X-C chemokine receptor 4 (CXCR4) was one of the most up-regulated genes in microglia 50. CXCR4 and its ligand stroma derived factor 1 (SDF1) are up-regulated in various cancers, and CXCR4 inhibition prevented metastasis formation 70. Studies of human breast cancer MCF-7 cells and the benign Madin-Darby canine kidney cells (MDCK) injected to brain slices demonstrated that microglia support invasion of breast cancer MCF-7 cells, but not the benign epithelial MDCK cells. The WNT inhibitor DKK2, as well as a CXCR4 inhibitor - AMD3100, reduced invasion of MCF-7 into the whole brain slice with similar efficacy 71. PI3K signaling was found active in the majority of breast cancer brain metastases. A systematic quantification of the PI3K pathway activity in breast cancer CNS metastases, using a reverse phase protein array, found a high PI3K activation in 62.5% brain metastatic tissues. PI3K signaling was activated in metastasis-promoting microglia/macrophages during CNS colonization. Treatment with buparlisib (BKM120), a pan-PI3K Class I inhibitor, reduced metastasis-promoting activity of microglia/macrophages 72.

Melanoma cells have a different propensity to colonize different organs. When two human melanoma cell lines were injected intracardially to immunodeficient mice: nude (nu/nu), NIH triple immunodeficient (TID: nu/nu, bg/bg, xid/xid) and severe combined immunodeficient (SCID) mice, MM-RU melanoma cells gave rise exclusively to lung metastases, whereas the MM-AN cells gave rise to lung and extra-pulmonary metastases. The metastatic lesions were circumscribed in all organs and had peripherally located macrophages, except for brain metastases, where a more invasive pattern along vasculature was observed 73. In immunocompetent animals, injected K-1735 melanoma cells formed metastatic lesions only in the brain parenchyma, whereas B16 melanoma cells and mixed B16 x K-1735 melanoma cells formed metastatic lesions only in the leptomeninges and ventricles. The difference in location of a metastatic tumor was likely due to the expression of transforming growth factor-beta 2 (TGF-β2) in melanoma cells: *TGF-β2* mRNA was highly expressed by the K-1735 cells, whereas the B16 cells or B16 x K-1735 cell mixes had low expression. Accordingly, manipulation of TGF-β2 expression in melanoma cells reduced metastasis to the brain parenchyma, but did not affect metastasis to the leptomeninges or ventricles 74.

After the intracranial transplantation of spontaneous melanoma brain metastasis cells to immunocompetent mice, activated astrocytes and microglia (stained with isolectin B4, ILB4) were recruited to the tumor-brain interface 75. Using CX3CR1-GFP transgenic mice allowed the visualization of the dynamic changes of microglia and macrophages during tumor growth of intracranially implanted melanoma cells through intravital imaging. Depletion of microglia and macrophages by treatment with PLX3397, an inhibitor of colony stimulating factor-1 receptor (CSF-1R), reduced the total number and mean size of the brain metastases by 83% and 65%, respectively. Microglia and macrophages from metastatic brains expressed MMP3 and treatment with PD166793, an MMP inhibitor, reduced the total number and mean size of the brain metastases by 50% and 53%, respectively 76. The results show the supporting role of tumor infiltrating microglia and macrophages.

Preclinical animal models well mimic the clinical course and neuropathology of human PCNSL and show pathological interactions between the malignant B cells, resident cell populations of CNS, and the associated immune infiltrates 77,78. Those interactions may foster aggressiveness of tumor cells and accelerate the fatal course of disease. A new player in interactions between microglia and cancer cells is Osteopontin (another name SPP1, small secreted phosphoprotein). Osteopontin is an activating factor for microglia and other immune cells. A gene coding for Osteopontin - *SPP1* is the most upregulated gene in PCNSL compared to non-CNS DLBCL 63,79. *SPP1* overexpression up-regulates invasiveness of B lymphoma cells in murine brain slices, promotes intracerebral invasion and dissemination of lymphoma cells. It increases the intracerebral lymphoma growth and shortens the survival in athymic mice. Mechanistically, these effects depend on intracellular Osteopontin (iOPN), which is encoded by one of the *SPP1* variants. The iOPN acts on transcription factor NFκB and causes transcriptional downregulation of the NF-κB inhibitors, *A20/TNFAIP3* and* ABIN1/TNIP1*, while secretory Osteopontin promotes receptor-mediated activation of NF-κB 63. It has been shown that glioma-derived Osteopontin is a potent inducer of microglia acting via integrin αvβ3 receptors at the microglial cells 80. It is likely that Osteopontin, produced by PCNSL cells, will act on microglia stimulating and inducing the pro-tumorigenic phenotype.

### 4.3. It is all about location - anatomical considerations in CNS metastases

The relative frequency of brain metastases in various anatomical regions of the brain differs: melanoma tends to metastasize to the frontal and temporal lobes, breast carcinoma to the cerebellum and the basal ganglia, large cell carcinoma of the lung to the occipital lobe and squamous cell carcinoma of the lung to the cerebellum 81. A recent study reported non-uniform distribution of metastatic brain lesions in breast and lung cancer patients. The lesions of NSCL cancer were preferentially located in the parieto-occipital lobes and cerebellum, while breast cancer lesions were in the cerebellum 82. CNS metastases generally locate in cortical regions, receiving blood supply from the most distal branches of large arteries i.e. anterior, middle and posterior cerebral arteries, and gray-white matter junction, probably due to slow blood flow velocity in those regions. Small intracranial melanoma metastases frequently occur at the interface between the cortex and leptomeninges 83. Application of high-resolution magnetic resonance imaging to such lesions demonstrated that deeper parenchymal extension of melanoma metastases occurs secondarily and suggests that the leptomeninges are a preferential site for establishment of melanoma metastasis 84. These findings suggest that different primary tumors could have propensities for different cerebral vascular areas and cerebral edema.

Leptomeningeal spread is a hallmark of hematological malignancies. It occurs in 5-15% of patients with non-Hodgkin's lymphoma and affects up to 10% of patients with acute lymphoblastic leukemia 39,40. Xenotransplantation of human ALL cells into immunodeficient NSG mice resulted in the infiltration leukemic cells exclusively to meninges and led to development of neurologic symptoms. CXCR4 inhibition (with CXCR4 antagonist AMD‐3100) impaired grafting of ALL cells to bone marrow, leukemia development and CNS infiltration 85. ALL cells migrate into CNS along vessels that pass directly between bone marrow and the subarachnoid space, bypassing the central circulation. The basement membrane of these vessels is enriched in laminin and its receptor - α6 integrin is expressed on most ALL cells. Interactions between α6 integrin and laminin mediated the migration of ALL cells towards the cerebrospinal fluid. Mice with ALL xenografts treated with a PI3Kδ inhibitor (which decreased α6 integrin expression on ALL cells) or specific α6 integrin-neutralizing antibodies, had significantly reduced transport of ALL cells along vessels 86.

Blood-brain barrier is crucial for maintaining CNS function by protecting neurons from minor disturbances in the systemic homeostasis. BBB is formed by tight junctions of endothelium, pericytes and astrocyte end-feet processes, which should shield CNS from circulating neoplastic cells. However, multiple processes are involved in migration of neoplastic cells through BBB. For instance, expression of Cathepsin S, a member of the cysteine cathepsin protease family, in primary tumor was associated with shorter time to development of brain metastases in immunocompromised mice injected with MDA-MB-231 cells. Of note, Cathepsin S was secreted by both cancer cells and microglia at the metastatic site and likely promoted BBB transmigration through cleavage of junctional proteins. Cathepsin S inhibition in both neoplastic and stromal cells reduced brain metastasis formation 87. Tumor-secreted micro-RNA were found to promote BBB transmigration. Breast cancer release exosomes containing miR-105 and miR-181c, and transfer their miRNAs to the endothelium which leads to downregulation of tight junctions and disrupts the BBB function 88,89. Interestingly, the brain microenvironment reciprocates - astrocyte-derived exosomes mediate transfer PTEN-targeting miRNA to metastatic breast cancer cells, leading to downregulation of this tumor suppressor. PTEN loss increases secretion of the chemokine CCL2, which recruits activated microglia 90. Strategies that could selectively open BBB in the brain metastasis are developed to increase pharmacological efficacy and limit CNS toxicity of the utilized therapy. A novel technique enhancing BBB penetration by pharmaceuticals is combination of focused ultrasounds with intravenously administered microtubules 91. This approach enhanced both small- and large-molecule drug delivery to the tumor in a murine model of breast cancer metastasis. The enthusiasm for BBB disruption techniques is dampen by the fact that widely used radiotherapy also has BBB-disruptive properties.

## 5. Strategies of targeting tumor-infiltrating microglia and macrophages in CNS metastases

Recent data on various compounds targeting brain macrophages in different CNS pathologies shed light on their contribution to shaping CNS immune microenvironment. This knowledge can be instrumental in development of potential therapeutics that can be used in a therapy of patients with CNS metastases. Glioblastoma (GBM) is a highly malignant, primary brain tumor which occurs frequently enough to be tested in randomized trials. The supportive role of microglia and macrophages in GBM is well understood and it is fairly well documented how these cells support tumor progression 16. Moreover, many strategies have been developed recently to target those cells in GBM and some of them have been successful in preclinical studies leading to clinical trials. The lack of drug effectiveness in GBM does not disqualify a specific treatment in CNS metastases and most of those drugs have advantage of crossing effectively BBB.

Experimental studies of CNS metastases in murine models provided a proof of concept for the concept to target tumor infiltrating microglia and macrophages. Application of the bisphosphonate clodronate to brain slice cultures injected with MCF-7 and 410.4 breast cancer cells reduced cancer cell invasion 50. A critical role of hypoxia in formation of tumor-permissive microenvironment was presented by adding nanoparticles releasing oxygen to culture of M2 macrophages in *in vitro* model, leading to decreased 4T1 cells mobility and invasiveness. Subsequent reduction of metastatic potential of those cells by the nanoparticles was confirmed in a murine model 92. Macrophage-colony stimulating factor (CSF-1) signaling through its receptor (CSF-1R) promotes the differentiation of myeloid progenitors into monocytes, macrophages, dendritic cells, and bone-resorbing osteoclasts. CSF-1/CSF-1R signaling facilitates recruitment and survival of TAMs within the tumor microenvironment in many cancers 93. Pexidartinib (PLX3397) is a novel, orally available, small molecule kinase inhibitor that blocks CSF-1R at an IC_50_ of 17 nM. The compound has other effects, it inhibits oncogenic, activated FLT3 (FLT3-ITK), interferes with SDF1-induced auto-phosphorylation of c-Kit protein at concentrations below 1 µM and inhibits differentiation of osteoclast precursors in the RANK-L and CSF-1 dependent manner 94. Treatment with pexidartinib reduced the total number and mean size of the brain metastases of intracranially implanted melanoma cells and improved the efficacy of adoptive cell therapy 95. Pexidartinib was effective in murine glioma models 96, but did not improve survival when compared with historical controls 97. A clinical trial of a single agent or combination of CSFR-1 and PD-1 inhibitors in advanced solid tumors is in progress 98.

**Figure 2:**
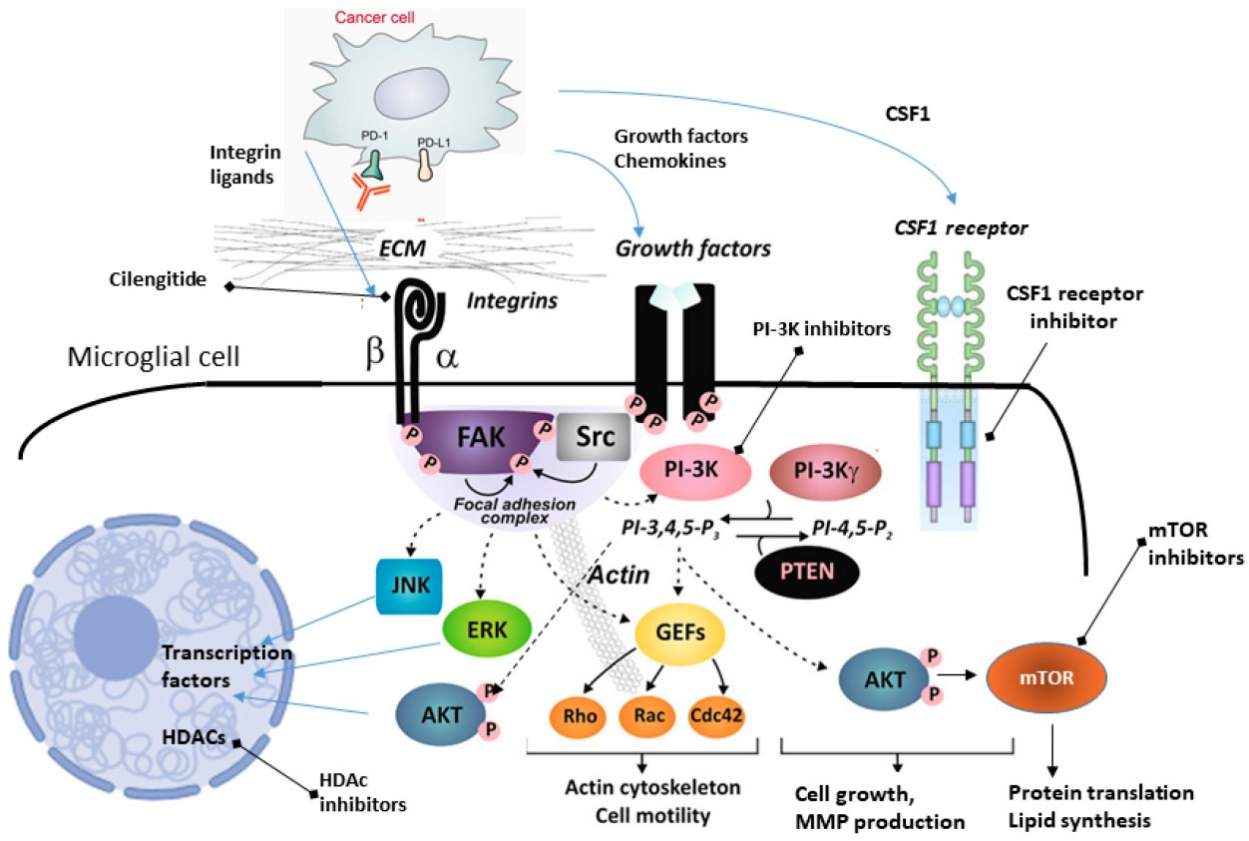
** A scheme illustrating intracellular signaling pathways that could be targeted with existing anticancer drugs in microglia in CNS metastases.** Activation of integrins stimulates a focal adhesion kinase (FAK) which forms a complex with Src family kinases, which initiates multiple downstream signaling pathways through phosphorylation of other proteins. Among those pathways are phosphatidylinositol 3-kinase (PI-3K), phosphatidylinositol-4,5-bisphosphate (PIP2), phosphatidylinositol-3,4,5-bisphosphate (PIP3), small G proteins (Rho, Rac, Cdc42), AKT/protein kinase B, ERK (extracellularly regulated kinase) and JNK (Jun N terminal kinase), and mTOR (mammalian target of rapamycin), all regulating different cellular functions. HDACs are histone deacetylases, epigenetic regulators that regulate histones, protein-DNA interaction, chromatin conformation, and transcription.

Several therapeutic strategies have been proposed to target glioma-associated microglia and macrophages. Those compounds target chemokines, metalloproteinases, integrins, tyrosine kinases, interleukin-6, CSF-1 and TGFβ functions, produced or acting both in cancer cells and stromal cells 99. Unfortunately, a few of those strategies were validated as microglia/macrophages targeting strategies and even fewer were explored in clinical trials. Figure 2 summarizes potential targets for therapeutics targeting microglia.

Of interest, cilengitide, a cyclic pentapeptide (cyclo-Arg-Gly-Asp-DPhe-NMe-Val) mimicking the RGD motif binding site, is a selective antagonist αvβ3 and αvβ5 integrin. It has bimodal action on blood vessels and brain tumor cells, inducing a cell death (*anoikis*, a cell death due to lack of anchorage) in the angiogenic endothelium and glioma cells. Activation of integrin signaling stimulates a focal adhesion kinase (FAK) which forms a complex with Src family kinases, which initiates multiple downstream signaling pathways through phosphorylation of other proteins. Among those pathways are phosphatidylinositol 3-kinase (PI-3K), phosphatidylinositol-4,5-bisphosphate (PIP2), phosphatidylinositol-3,4,5-bisphosphate (PIP3), AKT/protein kinase B, kinase ERK (extracellularly regulated kinase) and JNK (Jun N terminal kinase), and mTOR (mammalian target of rapamycin), all regulating different cellular functions (Figure 2). Interference in ECM or ligands binding to integrins, should affect all downstream signaling pathways.

Cilengitide showed some efficacy in the phase II CORE clinical trial and higher αvβ3 levels in tumor cells were associated with improved progression-free and overall survival 100. Microglia express high levels of αvβ3 integrins 80,100 and αvβ3 and αvβ5 integrins are expressed on macrophages 100. However, cilengitide failed to show superiority to temolozomide therapy in gliomas in the randomized phase III CENTRIC trial 101. Protein S (PROS1), a known ligand of TAM (Tyro-3, Axl, and Mer) receptor tyrosine kinase family 102 is secreted by tumor-associated macrophages/microglia and associates with an activated AXL kinase in mesenchymal glioma stem cells. Interestingly, PROS1 binds to AXL which leads to its activation and downstream targets of AXL, such as NF-κB, are activated, leading to expression of PD-L1 and subsequent cell growth. BGB324, a novel small molecule inhibitor of AXL, alone prolonged the survival of mice bearing human mesenchymal GBM cells, and combinatorial therapy with BGB324 and nivolumab prolonged the survival of mice bearing GBM tumors and increased cell death 103. AXL expression is an independent prognostic biomarker for survival outcome in brain metastases of NSCLC 104.

There is shortage of data regarding effectiveness of pharmaceuticals specifically affecting microglia/macrophages in brain metastases. Pexidartinib showed promising efficacy in breast cancer 105 and cilengitide was ineffective in melanoma and pancreatic cancer 106,107, but drugs were not explored for intracranial disease activity.

PI-3K is a common component of numerous signaling pathway activated by stimulation of integrins, receptors of growth factors, chemokines and cytokines in both cancer cells and microglia. Activating mutations in genes related to the PI-3K/AKT/mTOR-pathway occur in 43-70% of breast cancer brain metastasis patients. Buparlisib (BKM120) is a specific, oral pan-PI3K class I inhibitor. Treatment with BKM120 reduced metastasis-promoting activity of microglia/macrophages in animal models of breast cancer metastasis 72 and is currently under investigation in patients with breast cancer CNS metastases. The Akt-inhibitor GDC-0068 displayed antitumor activity towards breast cancer metastatic cells with activating mutations in the PI3K pathway *in vitro* and inhibited the growth of *PIK3CA*-mutant tumors breast cancer brain metastases *in vivo* 108*.* The inhibitors of mTOR are widely used in renal cell carcinoma, are safe in brain metastases 109 and are explored in combinations specifically for breast cancer 110. Whether some of their effects are mediated by inhibition of tumor infiltrating microglia is currently unclear. Notably, a recently developed platinum complex with selective activity against cells with active NFκB signaling was shown to act on activated macrophages 111.

There are indications of adding bevacizumab to existing treatment combinations in patients with various solid tumors brain metastases 112-114. VEGF signaling is involved in TAMs chemoattraction and proliferation 115, therefore some effects could be mediated by inhibiting microglia/macrophages. In a retrospective analysis of three clinical phase III trials, bevacizumab prevented or delayed the formation of brain metastases of non-small cell lung cancer, but not its metastases outside CNS 112. There was no effect on HER2 negative and HER2 positive breast cancer CNS metastases. In mice, treatment with bevacizumab inhibited formation of brain metastases by NSCLC cells and in improved overall survival 116.

A selective class IIa histone deacetylase (HDAC) inhibitor, TMP195, affected human monocyte responses to macrophage polarizing cytokines CSF-1 and CSF-2* in vitro* 117. *In vivo*, TMP195 treatment modulated macrophage phenotypes, altered the MMTV-PyMT breast cancer microenvironment, reduced tumor growth and pulmonary metastases. TMP195 induced the recruitment and differentiation of highly phagocytic, activated macrophages. When combined with chemotherapy regimens or T-cell checkpoint blockade, TMP195 treatment significantly reduced tumor burden 118.

There is a rationale to use immune checkpoint inhibitors in CNS metastases. Immune checkpoint inhibitors (anti-CLTA-4 antibodies and anti-PD-1/PD-L1 antibodies) potentiate host antitumor immune response and demonstrated an impressive clinical efficacy in advanced melanoma, metastatic kidney cancer, and metastatic non-small cell lung cancer, all malignancies that frequently cause brain metastases 27-29,38. Analysis of patients with brain metastases specimens showed that CD3+ TILs are present in 99.1% specimens and 56% cases had dense TIL infiltration. High density of CD3+ TILs was associated with longer median overall survival (15 months versus 6 months, respectively). The highest density of CD3+ TILs, CD8+ TILs, and PD-1-expressing T cells was found in melanoma brain metastases 48. Checkpoint inhibitors show activity in brain metastases of melanoma 119,120 and are at least safe in lung cancers 121,122. The combination of anti-PD-1 (nivolumab) and anti-CTLA4 (ipilimumab) therapy has a significant activity for CNS metastatic disease with a relatively low CNS-specific toxicity 119. In patients with melanoma brain metastases treated with the combination an intracranial response rate was 55% 119. The study of nivolumab in metastatic renal cell carcinoma (NIVOREN study) demonstrated safety and moderate efficacy of the drug 122. A phase II trial evaluating pembrolizumab (the anti-PD-1 monoclonal antibody) for patients with leptomeningeal carcinomatosis is ongoing 123. It is unfortunate that only a fraction of patients with untreated asymptomatic brain metastases (26%) can be enroll in such trials.

The effects on checkpoint inhibitors on accumulation of TAMs are not clear. Combination of PD-1 and CTLA-4 blockade reduced macrophage infiltration in B16 melanomas 103,124. In a melanoma model with an intracranial and extracranial (subcutaneous) B16 tumor, mimicking the coexistence of metastases inside and outside the brain, intracranial checkpoint inhibitors efficacy was observed only when extracranial tumor was present. The infiltration of microglia (CD11b+F4/80+CD45^low^) was increased following combined PD-1/CTLA-4 blockade and correlated with intracranial therapeutic efficacy. Simultaneous increase in CSF-1 within tumors was observed, which may explain increased microglia infiltration 125. Interestingly, repolarization of TAMs using exosome-mimetic nanovesicles derived from pro-inflammatory macrophages (M1NVs) led to release of pro-inflammatory cytokines and induction of antitumor immune responses *in vitro* and* in vivo*. Intravenous injection of M1NVs into tumor-bearing mice suppressed tumor growth and in combination with a PD-L1 antibody further reduced the tumor size 126. The current results of treatments with various inhibitors targeting microglia/macrophages are summarized in the Table 2.

Although, the existing data are fragmentary, it is likely that compounds affecting functions of microglia (and other brain macrophages) may affect brain metastases. As brain metastases were up to now investigated from a perspective of a specific metastatic site, it is difficult to assess whether the chemotherapeutics activity was mediated by local or systemic cells and factors.

## 6. Conclusions and perspectives

Breast, lung, and melanoma cancer cells exhibit a high tendency to invade CNS. The brain parenchyma and the leptomeninges/ventricular system represent two distinct microenvironments in CNS and certain cancer cells preferentially colonize those sites acquiring different features in the process. Survival of cancer cells and invasion of CNS are supported by different brain cell populations 143. Microglia and other non-parenchymal macrophages of CNS play important roles instigating and supporting metastases. Cancer cells up-regulate specific factors and hijack several mechanisms to polarize microglia and infiltrating peripheral macrophages into tumor supporting cells. The presented, yet fragmentary studies show important and unexplored roles of microglia and non-parenchymal, CNS resident macrophages in CNS metastases. There is a growing number of drugs specifically targeting brain tumor infiltrating microglia/macrophages (Figure 2, Table 2) that could be tested for a future use in therapy and/or prevention of CNS metastases. Those drugs have been pre-selected to pass blood brain barrier and their action on immune components of glioma microenvironment has been demonstrated. Many current approaches target similar oncogenic pathways that are activated in primary tumors, which does not account for metastatic cancer evolution and activation of unique, oncogenic mechanisms. Oncogenic pathways activated in extracerebral and brain metastases frequently differ, as do immune responses in CNS. Moreover, dysregulated pathways are different in each type of cancer. While is not yet clear if responses of brain macrophages are a tumor type specific, it has been demonstrated that activated brain macrophages exploit similar signaling pathways, therefore compounds targeting tumor supportive macrophages could be effective in CNS metastases of many cancer types. Certain pathways (i.e., PI3K signaling pathway) may be uniformly activated in different brain metastases and specific inhibitors could be effective in a large spectrum of CNS lesions. The recent “white paper” which presents the advances in basic science, translational, and clinical research in melanoma CNS metastasis and therapeutic management of patients, provides excellent recommendations for making significant clinical impact 144.

Leptomeningeal disease remains a unique challenge due to not fully understood pathobiology and a lack of cellular or animal models. Inclusion of cohorts of these patients to melanoma brain metastasis trials, or separate trials for these patients, will be important to move forward. Evaluating intrathecal therapies in these patients is justified. There is also a need to develop preclinical models of leptomeningeal disease to accelerate rational therapeutic development.

The presence of the blood-brain barrier is a limiting factor for the access of chemotherapeutics to metastatic lesions and several strategies to overcome this barrier have been developed. A number of small molecules crossing into the brain parenchyma, novel formulations of existing chemotherapeutics, and disruptive BBB techniques, including transcranial focused ultrasound coupled with intravenously delivered microbubbles, hyperosmotic agents (i.e. mannitol), radiation-induced permeability, have been reported 145. Basic research and preclinical studies concentrated specifically on brain metastases can lead to further improvements in systemic therapies for these patients.

## Figures and Tables

**Table 1:** Monocytic populations in the central nervous system and CNS metastases

Population	Markers	Brief description of functions	Role in metastases
Microglia	CD11b, CD68, CD115, CD206, CX3CR1, F4/80, Fcrls^++^, Iba1^+^, MerTK, P2ry12^++^, Siglec-H	In steady state: maintain homeostasis, exhibit a phagocytic activity, have low activity as antigen presenting cells	
	CD11b, CD45, CD64, CD68, CD115, CD163^+/-^, CD206, CX3CR1, F4/80, Fcrls^+^, Iba1^++^, P2ry12^+^	Upon activation: depending on stimuli polarization to an inflammatory of pro-tumorigenic phenotype: morphological changes, phagocytosis and antigen presentation, re-organization of ECM, cytokine and chemokine production	Tumor supporting phenotype degradation of ECM, modulating adaptive immune response, aiding in angiogenesis
CNS border-associated macrophages (BAMs)	CD11b, CD45, CD68, CD115, CD163, CD206, CX3CR1, F4/80	In steady state: maintain integrity of BBB. Upon pathological activation: modulate BBB integrity and vascular permeability, interact with circulating immune cells, produce reactive oxygen species.	Tumor supporting phenotype: facilitation of recruitment of cancer cells and immune cells via BBB
Infiltrating bone marrow-derived macrophages	CD11b, CD45, CD49D, CD64, CD68, CD115, CD163^+/-^, CD206, CCR2, F4/80, Ly6C	Infiltrate brain parenchyma mainly after breakdown of BBB; immunoregulatory and immunosuppressive functions; cytoprotective activity.	Tumor supporting phenotype: ECM remodeling, immune suppression, enhancement of angiogenesis.

**Table 2:** Molecular targets and therapeutics targeting microglia and macrophages

Molecular target	Affected processes	Pharmaceutical	Trials in glioma	Trials in brain metastases
CSF1R	Recruitment and enhancement of cancer invasion 4,103,126	Pexidartinib BLZ945	Phase II trial: lacking activity in comparison with historical controls 97	Limited; the phase I trial showed good activity in breast cancer 105, included in the adaptive phase II clinical trial 127.
αvβ3 and αvβ5 integrins	Microglia-assisted angiogenesis; polarization of microglia 128	Cilengitide	Phase III trial: lacking activity in comparison with temolozomide 101	Minimal to none clinical activity in metastatic melanoma and pancreatic cancer 106,107
Immune check point inhibitors	PD-1 or PDL1 CTLA-4	Nivolumab or/and ipilimumab	Phase III trial: lacking activity in comparison with bevacizumab 129	Nivolumab + ipilimumab combination is active in melanoma brain metastases 119,120, nivolumab is safe and active in NSCLC and renal cell carcinoma brain metastases 121,122
AXL kinase	AXL kinase regulates PD-1 expression 130	BGB324 alone or with Nivolumab	Inhibits glioma xenographs in pre-clinical trials 103	Not tested
mTOR	Polarization towards tumor-permissive phenotypes 131	Everolimus	Phase II trial: lacking activity in comparison with historical controls 132	Everolimus, lapatynib, and capecytabine combination had some activity in breast cancer brain metastases 110; safe and effective in renal cell carcinoma brain metastases 109
		Temsirolimus	Phase II trial: not superior to temolozomide but phosphorylation of mTORSer2448 can influence response 133	Safe and effective in renal cell carcinoma brain metastases 109
VEGF	TAMs chemotaxis and proliferation 115	Bevacizumab	Improves PFS but not OS in newly diagnosed glioblastoma 134	Encouraging results of various combinations in phase II trials with patients with brain metastases from NSCLC, breast cancer and colorectal cancer 112-114
		Regorafenib	Improves PFS and OS in previously treated glioblastoma 135	In phase III trials, regorafenib significantly increased OS, and PFS of patients with metastatic colorectal cancer 136
Histone deacetylase	Effects dependent on dose or cell types 137; TAMs polarization 116	Vorinostat	Improved OS when compared with historical results, a subgroup of patients with clear benefit 138	Promising results in preclinical models of triple-negative breast cancer 139
		Valproate	Increased survival in observational studies 140 and in comparison with historical cohorts 141	
		Romidepsin	Augmented temozolomide sensitivity in human glioma cells 139; lacking clinical activity 142	

PFS, progression free survival; OS, overall survival
